# The association of adult height with the risk of cardiovascular disease and cancer in the population of Sardinia

**DOI:** 10.1371/journal.pone.0190888

**Published:** 2018-04-20

**Authors:** Giovanni Mario Pes, Antonello Ganau, Eugenia Tognotti, Alessandra Errigo, Chiara Rocchi, Maria Pina Dore

**Affiliations:** 1 Department of Clinical and Experimental Medicine, University of Sassari, Sassari, Italy; 2 Sardinia Longevity Blue Zone Observatory, Ogliastra, Italy; 3 Department of Biomedical Sciences, University of Sassari, Sassari, Italy; 4 Baylor College of Medicine, Houston, TX, United States of America; CUNY, UNITED STATES

## Abstract

The relationship between body height and the risk of non‒communicable diseases such as cardiovascular disease and cancer has been the subject of much debate in the epidemiological literature. Concerns have recently arisen over spurious associations due to confounding factors like birth cohort, especially in the context of epidemiological transition. The population of Sardinia represents an interesting case study, as the average physical stature of inhabitants was the lowest recorded in Europe until a few decades ago. In this population we tested whether height is an independent risk factor for cardiovascular disease and cancer. We analysed the stature of 10,427 patients undergoing endoscopy for any reason, for whom a detailed clinical history of cardiovascular disease and/or malignancies had been documented. Poisson regression modelling was used to test the association between stature and disease risk. When patients were subdivided according to sex and height tertiles, the risk of cardiovascular disease proved significantly greater for subjects in the lowest tertile irrespective of sex (men: 1.87; 95%CI 1.41‒2.47; women: 1.23; 95%CI 0.92‒1.66) and smaller for those in the highest tertile (men: 0.51; 95%CI 0.35‒0.75; women: 0.41; 95%CI 0.27‒0.61). However, after adjusting the risk for birth cohort and established risk factors, it mostly resulted in non-significant values, although the overall trend persisted. Similar results were obtained for all-cancer risk (relative risk for men and women in the lowest tertile: 1.44; 95%CI 1.09–1.90 and 1.17; 95%CI 0.93–1.48, in the highest tertile: 0.51; 95%CI 0.36–0.72 and 0.62; 95%CI 0.47–0.81, respectively) as well as for some of the most common types of cancer. We concluded that the risk of developing cardiovascular disease and malignancies does not vary significantly with stature in the Sardinian population, after adjusting for birth cohort and more obvious risk factors.

## Introduction

Several retrospective and prospective studies suggested that the risk of cardiovascular (CV) disease and cancer, the leading causes of morbidity and mortality worldwide, is affected by adult height, although the magnitude and the precise direction of this association are quite controversial [1–3]. Observational epidemiological studies seem to support the notion that shorter individuals are at greater risk of developing CV disease, in both Western [1–9] and Asian countries [10], while they may show a relatively smaller risk of cancer [11–14]. Recently, the relationship between stature and disease risk was investigated through Mendelian randomization, an innovative methodology combining both genetic and epidemiological analysis, and whose results are less likely influenced by confounding and reverse causation [15]. This approach provided compelling evidence that genetically‒determined taller height is associated with significant lower risk of CV disease [16] and higher risk of colorectal cancer [17] in accordance with previous observational studies. However, if exposure is time‒variable, this approach may result in less accurate estimates of the causality [18]. Moreover, a small number of epidemiological studies have reported no association between short stature and CV disease [19–21], or even a positive association [22, 23]. Conversely, a possible link between being short and increased cancer incidence emerged from a minority of epidemiological studies [24, 25]. Although it is generally recognised that the mechanisms underlying these associations are not well understood [16], it is commonly believed that in addition to genetic factors, also dietary and endocrinological factors acting on bone growth during childhood may trigger the activation of pathogenetic mechanisms involved in the onset of chronic diseases [26]. A problem inherent in such epidemiological studies is that the average body height in a population is not stable over time but varies depending on early-life exposures, which may change during the course of history. The slow rise in average stature over the past century ‒ called *secular trend* ‒ was documented in nearly all developed populations, and was attributed to improvements in living standards and nutrition resulting from societal development over the last century. However, this period has also witnessed an increased risk of non‒communicable diseases, due to changes in nutrition habits and the impact of environmental and food carcinogens, to which both developed and developing societies were heavily exposed. As mentioned by Davey Smith et al. [2], in these conditions the true relationship between height and disease risk is likely to be rather distorted. In a population experiencing simultaneously an increase in average height and a rise in CV or cancer rate, spurious evidence may arise of a direct relationship between the two phenomena. Hence, epidemiological studies that neglect to account for this potential bias are likely to produce flawed results. Investigations aimed at testing the hypothesis of an association between height and non‒communicable disease risk should therefore consider birth cohort and socio-economic status (SES) [27] as modifying variables, assuming that older, short-statured generations may have been less exposed to risk factors for CV disease and malignancies than younger, taller generations. In addition, because of the marked sexual dimorphism in height and because men and women are exposed to different risk for non‒communicable diseases [28], sex should be considered both a potential confounder and effect modifier.

The population living on the island of Sardinia is particularly suited to an investigation of this kind for a number of reasons: (i) traditionally Sardinians were characterized by a shorter‒than‒average stature due to the harsh living conditions in the pre-industrial era, as well as the diffusion of endemic infectious diseases such as malaria [29, 30]; (ii) a marked secular trend in body height was documented in the Sardinian population [31], and recently the trend towards increased growth in height of Sardinian boys appears to have been the strongest in Europe with an increase of 1.2 cm per year [32]; (iii) an inverse relationship between height and longevity was reported in the male population of Central Sardinia with a 2‒years reduction in life span for taller people compared to their shorter peers [33]. These features of the Sardinian population can be interpreted in the light of evolutionary biology which postulates a trade‒off between growth and investment in reproduction [34]. More specifically, in a population historically characterized by poor environmental and nutritional conditions a stunting strategy may have represented an adaptive response resulting in greater fitness [30].

However, testing the stature‒disease risk hypothesis is feasible only when large databases are available. By gaining access to a large archive of patients in a health facility of Northern Sardinia, in this study we analysed the association between adult stature and CV and cancer risk while controlling for potential confounders such as birth cohort, sex and SES which affect both exposure and outcome, as well as established disease risk factors including smoking history, obesity, hypertension, diabetes, and hypercholesterolemia which act as mediators.

## Materials and methods

### Variables collection

A total number of 10,427 (6,388 women, 61.3%) unselected medical records of subjects from Northern Sardinia undergoing endoscopic procedures for any reason (dyspepsia, screening, surveillance programs, change in bowel habits and other conditions) from January 2002 to December 2016 at the University of Sassari’s Gastroenterology Unit, were chosen for the analysis. On admission for the endoscopic procedure, body height had been measured in centimeters for all study participants using a stadiometer, with the patient’s head aligned according to the Frankfurt horizontal plane, and body weight measured using an electronic scale with accuracy up to 0.1 kg [35]. Body mass index (BMI) was calculated as weight/height^2^; (kg/m^2^;). Demographic information was recorded in a computerized system, including sex, year of birth, smoking habits, history of definitive CV disease (acute myocardial infarction documented by diagnostic serum cardiac markers, typical history of effort‒induced angina, angiographically documented ≥ 70% narrowing of a coronary artery or lower limb artery) and cancer (all‒cancer and the most common site‒specific cancers), as well as established risk factors such as tobacco smoking, obesity, hypertension, diabetes mellitus and hypercholesterolemia.

### Ethics statement

A written informed consent was obtained from each patient included in the study. For minors, the written consent was provided by a parent. The study protocol was approved by the Ethics Committee: *Comitato di Bioetica*, *Azienda Ospedaliero-Universitaria di Sassari*, Italy (Prot No. 2477/2 CE, 2017).

### Statistical analysis

Categorical variables were described by using frequencies, continuous variables by using median and range. Study participants, stratified by sex, were further divided into three groups according to height tertiles. The middle tertile was taken as a reference group. Obesity was defined as BMI ≥ 30 kg/m^2^; [6]. On the ground of clinical history and laboratory tests, patients were classified as having type 1 or type 2 diabetes mellitus; however, for the purpose of the analysis, the two categories were pooled together as a binary variable since they can act both as risk factors regardless of their initial aetiology. Patients were stratified according to smoking habits (1 for not being smoker and 2 for being current or former smoker). The presence of high blood pressure was established according to 2013 ESH/ESC criteria [36] or if the patients was under anti‒hypertensive treatment. As for the lipid profile, patients were divided into two subgroups based on low-density-lipoprotein cholesterol levels at the time of endoscopy, higher or lower than the desirable value of 100 mg/dl recommended by the NCEP guidelines [37]. Socio-economic status was measured using current or past occupation as a proxy. Patients were initially divided into four groups: (i) graduated professionals; (ii), technicians and administrators (non-graduated); (iii) clerks and salesmen; (iv), semiskilled and unskilled workers, and uneducated shepherds and peasants; however, in the final analysis these groups were merged into two categories, i.e. high-SES including groups (i) and (ii), and low-SES including groups (iii) and (iv). In the case of multiple tests for the same patient within the given time period, only the last procedure was retained for the analysis. Part of this database was previously used for epidemiological and observational studies [38–40].

The average values or frequencies of demographic variables were calculated and their differences between tertiles were evaluated by one‒way ANOVA test for scalar variables or the χ^2^; test for categorical variables. The association between stature and presence/absence of CV disease or cancer as response variables was analyzed by Poisson regression modelling with height tertiles as an ordinal variable and controlling simultaneously for several known risk factors.

The first regression model (unadjusted) included only height tertiles; the subsequent model adjusted for variables considered as confounders (birth cohort and SES) or both confounders and effect modifiers (sex) and for the interaction term between sex and height; the last two models adjusted for mediator variables (tobacco smoking, obesity, hypertension, diabetes mellitus and hypercholesterolemia) and for both confounders and mediators (S1 and S2 Tables). Unadjusted and adjusted relative risk (RR) and their 95% confidence intervals (CIs) were calculated by exponentiating the regression coefficients. All statistical analyses were performed using SPSS statistical software (version 16.0, Chicago, IL, USA). P‒values lower than 0.05 were considered statistically significant.

## Results

Table 1 illustrates the characteristics of the study participants stratified according to sex and height tertiles. Among men the median height was 167.7 cm (lowest tertile 163 cm, middle tertile 168 cm, and highest tertile 172 cm). For women, median height was 157.7 cm (lowest tertile 154 cm, middle tertile 157 cm, and highest tertile 161 cm). In both men and women, the frequency of obesity was significantly higher in the lowest height tertile compared with the other two (p <0.0001). A similar pattern was found for other risk factors such as diabetes mellitus, hypertension, and hypercholesterolemia. As for smoking, a trend of decreasing frequency from the lowest to the highest tertile of height was detected in men, while in women the smoking frequency was unrelated to stature and always lower than among men. An inverse association between SES and stature was evident in both sexes.

**Table 1:** Characteristics of 10427 patients according to height categories.

Variables	Height tertiles					
	Men (n = 4039)			Women (n = 6388)		
	1 (< 165.0 cm)	2 (165.0‒169.9 cm)	3 (≥170.0 cm)	1 (< 156.6 cm)	2 (155.6‒158.9 cm)	3 (≥159.0 cm)
No. of patients	1346	1346	1347	2129	2129	2130
Median age (range)	69 (25‒91)	57 (25‒92)	42 (25‒90)	62 (25‒95)	58 (25‒91)	41 (25‒89)
Median birth cohort	1937	1951	1965	1945	1951	1965
BMI ≥ 30 kg/m^2^;, %	12.5	7.4	7.0	12.5	5.6	5.4
Diabetes mellitus, %	14.3	11.6	5.7	7.9	7.4	4.0
Hypertension, %	39.1	26.2	16.0	36.5	30.8	14.8
Smoking^a^, %	40.9	34.0	26.7	21.6	21.9	22.8
Hypercholesterolemia^b^, %	10.9	7.5	5.6	17.1	11.7	7.4
Low socio-economic status, %	59.2	48.1	47.5	69.9	65.1	53.1

^a^current or former smokers.

^b^defined as low-density-lipoprotein cholesterol ≥ 100 mg/dL according to the Third Report of NCEP Expert Panel on Detection, Evaluation, and Treatment of High Blood Cholesterol in Adults [37].

In Table 2 the relative risk for CV disease and all-cancers is reported by adjusting for sex, birth cohort, SES, obesity, diabetes, hypertension, smoke, and hypercholesterolemia by Poisson regression analysis. This analysis showed that he contribution of stature was not statistically significant neither for CV nor for cancer risk. No significant interaction between stature and sex was observed. The variation of the relative risk for CV disease and cancer in men and women according to height by stepwise selection of the groups of covariates (S1 and S2 Tables). Notably, the addition of birth cohort and SES to the regression model virtually suppressed the effect of stature on CV risk, although the overall trend remained, whereas controlling for the classical risk factors of CV disease affected the relative risk only slightly. Similar results were found for malignancies. Subjects of both sexes in the upper tertile of height showed significantly lower all‒cancers risk, and the effect disappeared only when adjusting for SES and birth cohort. A stature in the lowest height category was associated with an increased cancer risk, but it was significant in the unadjusted model and only in men.

**Table 2:** Poisson regression analysis including variables potentially associated with the occurrence of CV disease and cancer.

Variable	Cardiovascular disease		All‒cancers	
	RRs^a^ (95% CI^b^)	p‒value	RRs^a^ (95% CI^b^)	p‒value
Height, lowest tertile	0.93 (0.75‒1.16)	0.535	1.15 (0.91‒1.45)	0.245
Height, middle tertile	1.00	‒	1.00	‒
Height, highest tertile	0.92 (0.69‒1.23)	0.570	0.83 (0.67‒1.05)	0.116

^a^RR, adjusted relative risk.

^b^CI, Confidence Interval.

AIC, Akaike Information Criterion = 3254.9 and 4840.8 for CV disease and all-cancer, respectively.

Despite the small number of cases, a sub‒analysis was performed for the three most common site-specific tumours in Sardinia, namely colorectal, breast and prostate cancer [41]. In the case of colorectal and breast cancer the previous trend of an inverse association of tumour risk with stature was confirmed, which disappeared when controlling for birth cohort in the regression model. In the case of prostate cancer, this final adjustment revealed a significant positive association with short stature, but no definite conclusion was possible because of the small size of this cancer subset (S2 Table).

## Discussion

In today’s societies physical stature, especially in males, is largely perceived as a sign of health and longevity [6]. Nevertheless, many researchers have started to consider the recent rise in stature in European populations as an unwanted by‒product of the Industrial Revolution and the Western diet [22–23, 25]. It is commonly believed that there is a direct relationship between height and weight and that taller individuals are frequently overweight, which may entail a predisposition for chronic diseases. Although there is a strong evidence that obesity is harmful to long-term health and longevity, owing to excess visceral fat tissue [42], the relationship between stature and long‒term health is less clearly defined.

In this study we tested the hypothesis of an association between adult stature and the risk of developing non-communicable diseases, such as CV disease and cancer, in a population that is known as having the shortest average height in Europe [30], and in which an association between short stature and longevity had previously been reported [33]. Stratifying the study participants by sex and height tertiles, the relative risk of CV disease was significantly higher in the subjects in the lowest height category, irrespective of sex, and lower in those in the upper category. However, when the relative risks were adjusted for birth cohort and SES the risk was no longer significant, and was slightly affected by adjusting for the most common known risk factors like smoking, hypertension, hypercholesterolemia, obesity and diabetes. Similar results were found for all-cancer risk as well as for selected cancers like colorectal and breast cancer, with the exception of prostate cancer. These results suggest that the association of stature with crude risk of disease was essentially an artefact resulting from the failure to control for birth cohort. In fact, our data show that shorter individuals are likely to be born earlier, and to have a higher prevalence of diabetes, hypertension and dislipidemia, as reported by Korhonen et al. [43]. Thus, to isolate the independent role of stature in disease risk, it is necessary to take the generational structure of the population into account. Thus, cohort effect is a major potential confounder, as already reported by Jousilahti [24], and its impact should be evaluated in any future study on stature and the risk of non-communicable diseases in Sardinia. The overall pattern was sex‒independent, based on multivariate analysis, since the interaction between height and sex was not statistically significant.

The findings of our study, which do not support a significant relationship between stature and the risk of non‒communicable diseases, seem to contradict the results of Mendelian randomization studies showing a benefit of taller individuals in terms of the health and longevity [16, 17]. Actually, the Mendelian randomization approach works well only when exposure is not time‒varying. In the past, the average stature of Sardinian population has been heavily influenced by non‒genetic factors [30] and showed one of the strongest “secular trend” in stature. This could explain at least in part the discrepancy. One study reported an association between short stature and increased longevity in Sardinia [33]. However, as demonstrated by Lanari in the Sardinian male population of the 19^th^ century, the relationship between height and health ‒ the latter estimated by the probability of being considered fit for military service ‒ is non‒linear but displays an inverted‒U relationship [44]. Consequently, to explore the relationship between stature and survival, any method based on the assumption that there is a linear relationship between the response variable and predictors should be applied cautiously. In addition, the relationship between short stature and longevity does not hold for other long‒lived populations outside Sardinia. For example, the elderly population of Nicoya, Costa Rica, with one of the lowest recorded mortality levels in the world, is also significantly taller than the other Costa Ricans [45].

Our study has some strengths and limitations. The analysis was conducted with a large database, body height was measured accurately and not just self‒reported as in most studies, and the analysis covered all the birth cohorts in the last century. However, our analysis may not have included unidentified factors affecting disease risk, and subjects with specific health problems affecting stature were not excluded. Although socio-economic status was included among the covariates of the regression models, it reflected the later phases of existence, and no information was available on the socio-economic status of childhood, the only one that might affect stature. Finally, the population sample was recruited from Northern Sardinia and the results obtained might not be extended to populations of different ethnic origin. Even with these limitations, this study lays the foundations for a future in-depth investigation into the relationship between stature and survival in Sardinia.

## Conclusion

In conclusion, the results of this study provided evidence that also in the Sardinian population the risk for cardiovascular disease and most malignancies does not vary significantly with body height after adjusting for birth cohort and more obvious risk factors.

## Supporting information

S1 Table Unadjusted and adjusted RRs for CV disease according to height tertiles in men and women. (PDF)

S2 Table Unadjusted and adjusted RRs for all cancers combined and some site-specific cancers according to height tertiles. (PDF)

S1 File Minimal general data. (XLSX)
