# Supplementary material for: The association of adult height with the risk of cardiovascular disease and cancer in the population of Sardinia
Source: PLoS One. 2018 Apr 20;13(4):e0190888. doi: 10.1371/journal.pone.0190888 (PMC5909893; doi:10.1371/journal.pone.0190888)
Supplement: S1 Table — (PDF) [file pone.0190888.s001.pdf]

Table 1. Unadjusted and adjusted RR for CV disease according to height tertiles in men and women.

CV disease	Height tertiles					
	Men (n=4039)			Women (n=6388)		
	1 (< 164.0 cm)	2 (164.0–168.4 cm)	3 (≥168.5 cm)	1 (< 155.5 cm)	2 (155.6–158.1 cm)	3 (≥158.2 cm)
No. of patients	1346	1346	1347	2129	2129	2130
No. of cases	142	76	39	103	84	35
Unadjusted RR	1.87 ** (1.41–2.47)	1.00	0.51 ** (0.35–0.75)	1.23 (0.92–1.66)	1.00	0.41 ** (0.27–0.61)
Adjusted RR[a]	1.02 (0.74–1.41)	1.00	0.89 (0.59–1.33)	0.86 (0.63–1.18)	1.00	0.93 (0.62–1.41)
Adjusted RR[b]	1.67 * (1.23–2.27)	1.00	0.61 * (0.40–0.92)	1.09 (0.81–1.48)	1.00	0.53 * (0.35–0.80)
Adjusted RR[c]	1.01 (0.73–1.43)	1.00	0.87 (0.57–1.34)	0.84 (0.62–1.15)	1.00	0.92 (0.61–1.40)

[a]Adjusted for potential confounders (socio–economic status and birth cohort); [b]Adjusted for known mediator variables (smoke, hypertension, BMI ≥ 30 kg/m², hypercholesterolemia, diabetes); [c]Adjusted for both confounders and mediators; * p<0.05; ** p<0.001

Relative to the middle tertile as reference group, the crude CV risk was significantly greater in subjects in the lowest tertile irrespective of gender sex (men: 1.87; 1.41–2.47; p<0.0001; women: 1.23; 0.92–1.66; n.s.) and smaller in those in the highest tertile (men: 0.51; 0.35–0.75, p=0.006; women: 0.41; 0.27–0.61; p=0.006) (Table 1).
