# Supplementary material for: The association of adult height with the risk of cardiovascular disease and cancer in the population of Sardinia
Source: PLoS One. 2018 Apr 20;13(4):e0190888. doi: 10.1371/journal.pone.0190888 (PMC5909893; doi:10.1371/journal.pone.0190888)
Supplement: S2 Table — (PDF) [file pone.0190888.s002.pdf]

Table 2. Unadjusted and adjusted RRs for all cancers combined and some site-specific cancers according to height tertiles.

	Height tertiles					
	Men (n=4039)			Women (n=6388)		
	1 (< 164.0 cm)	2 (164.0–168.4 cm)	3 (≥168.5 cm)	1 (< 155.5 cm)	2 (155.6–158.1 cm)	3 (≥158.2 cm)
No. of patients	1346	1346	1347	2129	2129	2130
All cancers						
No. of cases	130	93	49	166	143	91
Unadjusted RR	1.44 * (1.09–1.90)	1.00	0.51 ** (0.36–0.72)	1.17 (0.93–1.48)	1.00	0.62 ** (0.47–0.81)
Adjusted RR[a]	0.95 (0.70–1.28)	1.00	0.75 (0.52–1.09)	1.07 (0.84–1.35)	1.00	0.88 (0.66–1.17)
Adjusted RR[b]	1.35 * (1.02–1.78)	1.00	0.55 ** (0.38–0.79)	1.13 (0.90–1.43)	1.00	0.64 * (0.49–0.84)
Adjusted RR[c]	0.94 (0.70–1.28)	1.00	0.76 (0.52–1.10)	1.05 (0.83–1.33)	1.00	0.88 (0.66–1.17)
Colorectal cancer						
No. of cases	23	19	8	24	15	10
Unadjusted RR	1.21 (0.66–2.24)	1.00	0.42 * (0.18–0.96)	1.51 (0.80–2.84)	1.00	0.62 (0.28–1.38)
Adjusted RR[a]	0.74 (0.38–0.45)	1.00	0.66 (0.28–1.54)	1.24 (0.65–2.37)	1.00	1.10 (0.49–2.51)
Adjusted RR[b]	1.13 (0.61–2.09)	1.00	0.45 (0.19–1.03)	1.38 (0.73–2.63)	1.00	0.69 (0.31–1.54)
Adjusted RR[c]	0.73 (0.37–1.43)	1.00	0.65 (0.28–1.53)	1.19 (0.62–2.28)	1.00	1.13 (0.50–2.56)

Breast cancer						
No. of cases	–	–	–	82	71	37
Unadjusted RR				1.15 (0.84–1.59)	1.00	0.52 (0.35–0.77) **
Adjusted RR[a]				1.17 (0.85–1.60)	1.00	0.51 (0.34–0.76) **
Adjusted RR[b]				1.07 (0.78–1.48)	1.00	0.69 (0.46–1.05)
Adjusted RR[c] §						
Prostate cancer						
No. of cases	16	15	6	–	–	–
Unadjusted RR	1.06 (0.53–2.16)	1.00	0.40 (0.15–1.03)			
Adjusted RR[a]	0.40 (0.18–0.87) *	1.00	0.78 (0.30–2.02)			
Adjusted RR[b]	0.93 (0.45–1.92)	1.00	0.44 (0.17–1.14)			
Adjusted RR[c]	0.38 (0.17–0.85) *	1.00	0.76 (0.29–1.98)			

[a]Adjusted for potential confounders (socio–economic status and birth cohort); [b]Adjusted for known mediator variables (smoke, hypertension, BMI ≥ 30 kg/m², hypercholesterolemia, diabetes); [c]Adjusted for both confounders and mediators; * $p<0.05$; ** $p<0.001$
